# Short‐term effects of SAFE early intervention approach in infants born preterm: A randomized controlled single‐blinded study

**DOI:** 10.1002/brb3.3199

**Published:** 2023-08-03

**Authors:** Umut Apaydın, Ramazan Yıldız, Ayşe Yıldız, Şebnem Soysal Acar, Kıvılcım Gücüyener, Bülent Elbasan

**Affiliations:** ^1^ Faculty of Health Sciences, Department of Physiotherapy and Rehabilitation Karadeniz Technical University Trabzon Turkey; ^2^ Faculty of Health Sciences, Department of Physiotherapy and Rehabilitation Gazi University Ankara Turkey; ^3^ Faculty of Medicine, Department of Pediatrics, Section of Pediatric Neurology Gazi University Ankara Turkey

**Keywords:** early intervention, environmental enrichment, infant, premature

## Abstract

**Objective:**

Recent literature suggests that goal‐oriented and family‐based interventions in enriched environment have a beneficial effect on neuromotor and cognitive development. We aimed to examine the short‐term effects of SAFE (**S**ensory strategies, **A**ctivity‐based motor training, **F**amily collaboration, and **E**nvironmental Enrichment) early intervention approach on motor, cognitive, speech and language, and sensory development in preterm infants.

**Methods:**

The study's sample population consisted of 24 preterm infants with corrected ages between 9 and 10 months. Infants in the control group participated in the family training program in accordance with the neurodevelopmental therapy principles (NDT). Infants in the treatment group were included in the family training program according to the principles of the SAFE Early Intervention Approach. Affordances in the Home Environment for Motor Development‐Infant Scale (AHEMD‐IS), Test of Sensory Functions in Infants (TSFI), Canadian Occupational Performance Measure (COPM), and Bayley Scales of Infant and Toddler Development III (Bayley III) were used to evaluate infants in both groups before and after 10 weeks of treatment (AHEMD‐IS). The Depression, Anxiety, Stress Scale Short Form was used to assess the parents’ mental health (DASS‐SF).

**Results:**

The interaction effects (time × group) revealed significant differences for Bayley‐III cognitive and language scores, TSFI total score, and AHEMD‐IS total score in favor of the SAFE group (*p* < .05). However, there were no differences in Bayley‐III motor composite score, COPM Performance score, and COPM Satisfaction score between the interaction effects (time × group) of the groups (*p* > .05).

**Conclusions:**

SAFE early intervention approach improved cognitive, speech and language, sensory outcomes and provide enriched home environment in all domains when compared to NDT‐based home program. SAFE is a promising novel early intervention approach for preterm infants.

## Abstract 2

Our article examines the short‐time effects of newly developed early intervention approach that is used for premature infants.

## 1 INTRODUCTION

Infants with a history of premature birth and low birth weight are at risk of developing retardation (Blauw‐Hospers et al., 2007). These infants may experience motor, cognitive, and behavioral problems compared to their typical peers (Bhutta et al., 2002). While technological advances are resulting in increased survival rates, 50% of these infants may experience developmental delays in the motor, cognitive, and behavioral areas. Therefore, early intervention is crucial to support neuropsychomotor development in early childhood.

Many intervention programs have been described in preterm infants. The most widely used of these is neurodevelopmental therapy (NDT). However, the results of the studies on the effects of NDT on motor development are contradictory (Damiano, 2013; Te Velde et al., 2022). Although minimal changes occur with NDT in infancy, recent systematic reviews on the effect of early intervention in high‐risk infants have shown that NDT does not improve neurodevelopmental outcomes (Blauw‐Hospers & Hadders‐Algra, 2005; Blauw‐Hospers et al., 2007: Spittle et al., 2012; Te Velde et al., 2022). Also, passive techniques like stretching and passive range of motion exercises are generally provided with “hands‐on” techniques. However, these techniques hinder the baby's own activities, kinesthetic sensation, and motor learning processes and limit their experiences of different movement strategies and their variations. “Just‐right challenge” in various conditions gives the child the opportunity to explore the environment in their daily activities; thus, the child learns to adapt to the conditions of daily life (Dirks et al., 2011). It is also known that exposure to movement variations causes an increase in the child's motor repertoire (Hadders‐Algra, 2000). For these reasons, positioning, stretching, or passive range of motion exercises have been abandoned in recent years, and goal‐oriented activities and environmental enrichment in daily routines and natural context have been taken into account in treatment protocols (Morgan et al., 2015; Law et al., 2011). These programs are home‐based practices in the child's natural environment, in which healthcare professionals take part in the intervention with coaching, supporting, and families play an important role in early childhood intervention (Dirks & Hadders‐Algra, 2011; Holt & Mikati, 2011).

In recent years, as the effects of “environmental enrichment” on brain development have been proven, the focus of early intervention has shifted to enriched environmental interventions to support psychomotor and cognitive development (Morgan et al., 2013). In this context, the Goal, Activity, Motor Enrichment (GAME) has been developed, which includes goal‐oriented intensive motor training, family education, and enrichment of the environment. The protocol is based on the concepts of dynamic systems theory and motor learning (Green, 2014; Morgan et al., 2014). According to this protocol, families set goals to support their child's development. Families collaborate with therapists in setting these goals. Goals relate to motor development, but also include conditions that affect development, such as sleep and nutrition (Morgan et al., 2014).

Another early intervention approach developed in the latest years is Supporting Play, Exploration, and Early Development Intervention (SPEEDI). SPEEDI is an approach designed to increase limited abilities through early experience. In this approach, environmental enrichment, presenting motor and sensory learning principles and providing opportunities to develop motor and cognitive skills with cooperation between family−infant−therapist, plays an important role. In this approach, parents are taught to encourage infants’ movements through environmental enrichment rather than imposing passive movement experiences on infants (Dusing et al., 2020; Dusing et al., 2018).

Recently, positive developmental results have been shown in preterm and infants at risk with early intervention approaches using principles like family‐oriented, home‐based, and environmental enrichment. As the positive effects of these approaches on the motor, sensory, socioemotional, speech and language, and cognitive development have been proven, each country has started to develop its own early intervention protocols within this framework like GAME and SPEEDI (Dusing et al., 2020; Morgan et al., 2014) according to their resources and needs. In our country, generally techniques, such as passive stretching, positioning, and passive range of motion exercises, are widely used in the intervention for infants at risk. Additionally, therapists frequently use hands‐on techniques in order to facilitate motor milestones like crawling and assist appropriate motor development. Infants are not allowed to experience movement and its variations on their own body by trial‐and‐error with kinesthetic sensation. Similarly, training and collaboration with the family are not considered as important aspects of early childhood intervention in their natural context. It is an inevitable fact that our country also needs an early intervention approach that is suitable for its financial, cultural, and socioeconomical context. A novel intervention approach that may be implemented to babies between the ages of 0 and 24 months was designed within the context of this study. The principles of this early intervention approach include sensory strategies, activity‐based motor training, environmental enrichment, and family collaboration. The SAFE approach is based on neuronal group selection theory (NGST), dynamical systems theory (DST), and motor learning principles. The approach also includes ecological model principles in its practical applications at the natural context. The English initials of these guiding principles were used to establish the name of the early intervention program (S: Sensory Strategies, A: Activity‐Based Motor Training, F: Family Collaboration, E: Environmental Enrichment). Establishing the theoretical and practical framework of the SAFE early intervention approach, created by the Pediatric Physiotherapy Rehabilitation Department at Gazi University, was the goal of this study. In addition, it was aimed to examine the effects of this approach on the sensory, motor, cognitive, and language development in premature infants with corrected ages of 9–10 months.

## 2 MATERIALS AND METHODS

### 2.1 Participants

Thirty‐eight infants born premature and their families were invited to this study. The study was conducted at Pediatric Rehabilitation Unit in Faculty of Health Sciences at Gazi University. The inclusion criteria were: (1) being born before 37 weeks of GA; (2) having a history of Neonatal Intensive Care Unit (NICU) of 15 days or more; (3) corrected age of 9–10 months; and (4) willingness of family to participate in this study. Exclusion criteria were: (1) having a history of a congenital anomaly or systemic disease; (2) medical conditions which prevent active participation in therapy (such as dependence on oxygen); and (3) living out of reach of the research team for home visits. The presence or absence of fidgety movements was not taken into consideration and there were videos for the fidgety period of 10 babies. All of these babies had fidgety movements.

Based on the similar study (Morgan et al., 2014), we calculated the sample size (*n* = 12 for each group) using power analysis program (G*power version 3.1.9.2, Axel Buchner, Universität Kiel). The power set at 0.8 and alpha at 0.05 for detecting moderate‐sized effects (*η* ^2^ *p* = .06) (Cohen, 1988).

### 2.2 Study protocol and timeline

Families of newborns provided written, informed consent. The study was approved by the Gazi University Clinical Research Ethics Committee. Also, this study was registered at Clinical Trials.gov (NCT04889846). The study started in October 2019. However, with the outbreak of the Covid‐19 pandemic in December 2019, there were losses in follow‐up, and the study was completed in October 2021.

### 2.3 Study design

**FIGURE 1:**
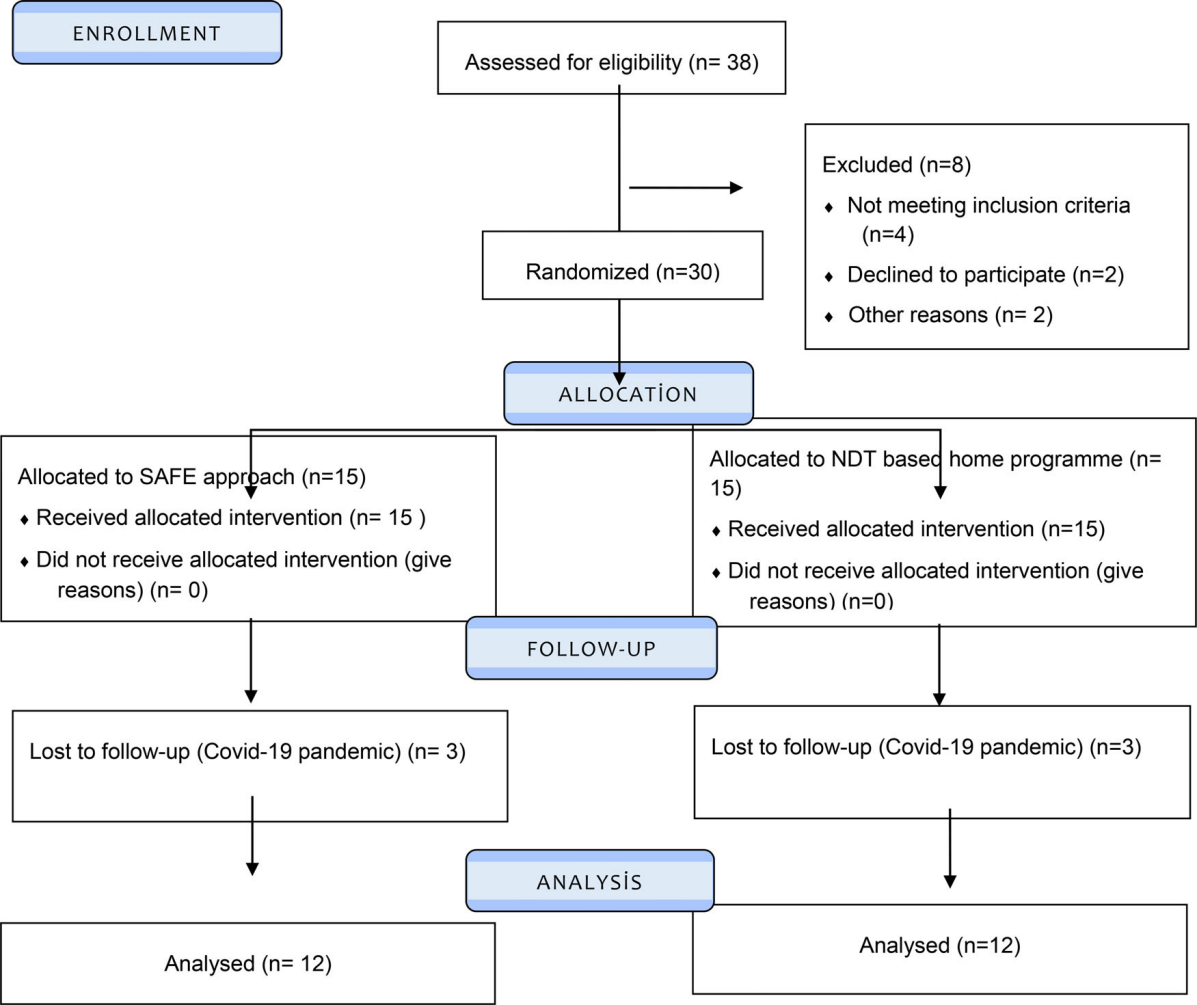
CONSORT flow diagram illustrating the infants participating in the study.

A single‐blinded, 1:1 randomized, and controlled experiment was used to construct the investigation. Thirty infants were eligible for inclusion. The minimization method was used to balance the gender. The participants were randomly assigned to the SAFE early intervention approach (SAFE, *n*: 15) and the NDT program (NDT, *n*: 15) (Scott et al., 2002). Random allocation software was used for randomization. The researcher who was in charge of randomization was not involved in gathering or analyzing data. Two researchers who were blinded to the randomization and the history of the babies assessed the infants at baseline (T1) and 10 weeks later (T2). The Consolidated Standards of Reporting Trials (CONSORT) flow chart in Figure 1 presents the research design.

### 2.4 Intervention

#### 2.4.1 SAFE

SAFE early intervention approach is family‐centered and focuses on activity‐based motor training and sensory strategies in an enriched environment. The theoretical and clinical framework of the intervention was developed in the Pediatric Physiotherapy and Rehabilitation Department of Faculty of Health Sciences at Gazi University. The early intervention approach is carried out in the child's natural context and collaboration is established between the physiotherapist and family/caregivers to implement the program. The SAFE approach is based on NGST (Hadders‐Algra, 2000), DST (Thelen, 1995), and motor learning principles. The approach includes also ecological model principles in its practical applications (Bronfenbrenner & Morris, 2007). Detailed information about the approach is given in Supplement A.

#### 2.4.2 NDT‐based home program

NDT principles are used in physiotherapy and rehabilitation program of preterm infants at risk in our country which was also implemented to the control group in our study. In addition, comments were offered to families for cognitive, speech, and language development along with the NDT program according to their needs. Detailed information about the NDT program is given in Supplement A.

At least two home visits were made for both the SAFE early intervention group and the control group. Three physical therapists conducted the home visits. One of these physical therapists had 10 years of experience, while the others had 8 years of experience in pediatric rehabilitation and early childhood intervention. Additional recommendations were made to families during these visits. Families’ adherence to the program was checked via phone calls or the WhatsApp program.

### 2.5 Measurements

#### 2.5.1 Motor, cognitive, and language development

Bayley Scales of Infant and Toddler Development, Third Edition (Bayley‐III) is frequently used to assess the cognitive, language, gross, and fine motor development of children between the ages of 1 and 42 months. The Bayley‐III's cognitive scale evaluates a range of developmentally appropriate tasks, including counting, solving puzzles, matching colors, and playing different kinds of games. The Bayley‐III motor scale measures both gross and fine motor skills, including sitting, crawling, standing, leaping, and climbing stairs. Fine motor skills include visual tracking, reaching, and gripping. The Bayley‐III language scale assesses spelling, body language, object identification, vocabulary use, plural suffixes, and verb conjugations. Each section's items are given a score between 0 and 1 (may perform the desired skill) (may not do the desired skill). Scaled scores ranging from 1 to 19 points are created from the raw scores acquired for each item. The scaled score is then used to create a composite score. The range of the composite scores is 40–160. Better development is indicated by a higher score. A composite score of less than 85 in any area of development indicates a developmental delay in that area (Bayley & Bayley, 2006). Bayley‐III was administered by a blind psychologist to the groups with 20 years of experience in the field. The Bayley‐III was carried out at GaziUniversity Faculty of Medicine, Department of Pediatrics, Psychomotor evaluation room. Appropriate environmental conditions (temperature, lighting, sound) were provided in the evaluation room.

To evaluate sensory abilities, the Test of Sensory Functions in Infants (TSFI) was used. Infants between the ages of 4 and 18 months are evaluated for sensory processing and responsiveness using the TSFI. Adaptive motor response, tactile deep pressure response, visual‐tactile integration, vestibular stimulation, and oculomotor test are the five subtests that consist of the TSFI. The overall score ranges from 0 to 49. Children between the ages of 10 and 12 months should score between 44 and 49 to show adequate sensory function, between 41 and 43 to suggest a risky condition, and between 0 and 40 to indicate a sensory integration problem (DeGangi & Greenspan, 1989; Jirikowic et al., 1997). In this study, TSFI was performed by an evaluator blinded to the groups in a room with appropriate environmental conditions.

#### 2.5.2 Function

Canadian Occupational Performance Measure (COPM) has been widely used in goal‐oriented studies in premature infants (Morgan et al., 2016). In this study, COPM was used to identify functional goals. In this way, the goals of families were given priority for the development of infants. Perceptions of families regarding the performance of infants on the set goals were determined. In addition, parents’ satisfaction with the baby's current abilities was evaluated. The activities that the infants had problems with (e.g., not being able to touch rough surfaces, crawling, cruising, standing independently, and fear of noisy environments) were determined by the families before the treatment. Performance and satisfaction scores of families were determined for these activities. After the treatment, the related activities were re‐evaluated and scored.

#### 2.5.3 Home environment

Home enrichment was assessed using the Affordances in the Home Environment for Motor Development‐Infant Scale (AHEMD‐IS). AHEMD‐IS evaluates indoor space, outdoor space, toys, and equipment variety that support motor development. The scale comprises 41 items. For infants 3–12 months of age, the first 32 questions are answered. All items are answered in infants aged 12–18 months. No and yes responses are scored as 0 and 1 (0: no, 1: yes). The remaining questions are scored as 0, 1, 2, and 3. (0: never, 1: sometimes, 2: almost always, 3: always). For infants less than 12 months of age, a total of 66 points is obtained. For infants over 12 months of age, a total of 93 points is obtained. A higher score indicates a better enrichment of the home environment (Caçola et al., 2011; Caçola et al., 2015). In this study, the AHEMD‐IS test was given to the family and they were asked to complete this test. The raw score was used in the analysis.

#### 2.5.4 Parent mental health

The Depression, Anxiety and Stress Scale‐Short Form (DASS‐21) is a questionnaire that assesses individuals’ depressed, anxious, and stressful conditions. DASS‐21 was used to evaluate the parent's mental health. It is effective in detecting patients suffering from depression and anxiety disorders (Ng et al., 2007). In addition, due to the number of items, it enables the severity of three psychological states to be measured in a short time. The 21‐item DASS‐21 consists of 7 items about depression, 7 items about anxiety, and 7 items about stress. The questions are scored as 0, 1, 2, and 3 (0: never, 1: sometimes, 2: often, 3: always). A low score indicates a good psychological state (Lovibond & Lovibond, 1995; Saricam, 2018).

The data about sensory abilities, functions, home environment, and parent mental health were collected in Pediatric Physiotherapy and Rehabilitation Department of Faculty of Health Sciences at Gazi University. Appropriate environmental conditions (temperature, lighting, sound) were provided in the evaluation room.

#### 2.5.5 Dose of intervention

Families were encouraged to keep a logbook and note how long each activity occurred. Information was obtained from the families every week, and at the end of the treatment, the logbook were taken from the families and the intervention times were recorded.

### 2.6 Statistical analysis

Data analysis was done using SPSS Software 22 (SPSS Inc.). Data normality was assessed using analytical techniques like the Shapiro−Wilk test and visual techniques like histograms. The data for T1 (Pre‐treatment) were compared across groups using an independent sample *t*‐test or a Mann−Whitney U test. Within group T1–T2 alterations, effect sizes were determined in line with Cohen's norms. Little effect (0.2), moderate effect (0.5), or strong effect (0.8) were recorded for Cohen's *d* results (Cohen, 1988). Two‐way repeated‐measures ANOVA was used to investigate the time × group interaction. When significant interactions were identified, post‐hoc pairwise analysis was performed. Partial eta‐square (*η* ^2^ *p*) was interpreted as effect size as small (*η* ^2^ *p* = .01), medium (*η* ^2^ *p* = .06), and large (*η^2^p* = .14) (Cohen, 1988).

## 3 RESULTS

### 3.1 Participants

A total of 38 infants and their families were eligible for this study. Of the 38 infants, four did not meet the inclusion criteria, two families refused to participate in the study, and the two families’ home was too far to make a home visit. Thus, 30 out of 38 were evaluated and allocated to the groups. There was one pair of twins in both the NDT and the SAFE group. During the 10‐week follow‐up period, three infants from the NDT group and three infants from the SAFE group did not participate in the final evaluation due to the Covid‐19 pandemic. The analysis was performed per protocol on the infants included in the study (Figure 1).

Table 1 provides information about the participants’ demographic and medical characteristics, and no differences were observed between groups except the gestational age (*p* > .05).

**TABLE 1:** Characteristics of the participants.

Infants characteristics		SAFE	NDT	*p*	95% CI
Age	Enrolment corrected age (weeks) mean (SD)	37.1 (1.1)	38.2 (0.9)	.225	−7.56 to 22.83
Sex	Male, *n* (%)	5 (41)	5 (41)	1	NA
Weight	Birth weight (gr) mean (SD)	1331 (445)	1653 (448)	.092	−699.87 to 56.54
Gestational age	Birth gestational age (weeks) mean (SD) 26–28 weeks, *n* (%) 28–32 weeks, *n* (%) 32–34 weeks, *n* (%)	29.2 (2.2) 5 (41.7) 4 (33.3) 3 (25)	31.1 (2.3)^*^ 2 (16.7) 4 (33.3) 6 (50)	**.045**	−3.80 to −0.04
NICU stay	NICU stay (days) mean (SD)	49 (26)	36 (25)	.224	−8.61 to 34.78
Family characteristics	Maternal age (years)	30.9 (5)	30.1 (5.4)	.729	−3.68 to 5.18
	Mother's education Secondary school *n* (%) High school *n* (%) Bachelor degree *n* (%) MSc, PhD *n* (%)	0 (0) 1 (8.3) 10 (83.3) 1 (8.3)	1 (8.3) 5 (41.7) 6 (50) 1 (8.3)	.079	−0.068 to 0.079

Abbreviations: MSs, master of science; NDT, neurodevelopmental treatment‐based home program; NICU, neonatal intensive care unit; PhD, doctor of philosophy; SAFE, SAFE Early Intervention Approach; SD, standard deviation.

^*^ *p* < .05.

### 3.2 Data normality

We employed parametric statistics since the Bayley‐III, TSFI, COPM, and AHEMD‐IS were normally distributed at both time points. The DASS‐21 total scores at baseline and follow‐up, as well as all of its subscales, were not normally distributed; as a result, we utilized nonparametric statistics to analyze the DASS‐21. Similar to the dosage of the intervention, which was not normally distributed, we conducted our analyses using nonparametric statistics.

### 3.3 Effects of SAFE early intervention approach

**FIGURE 2:**
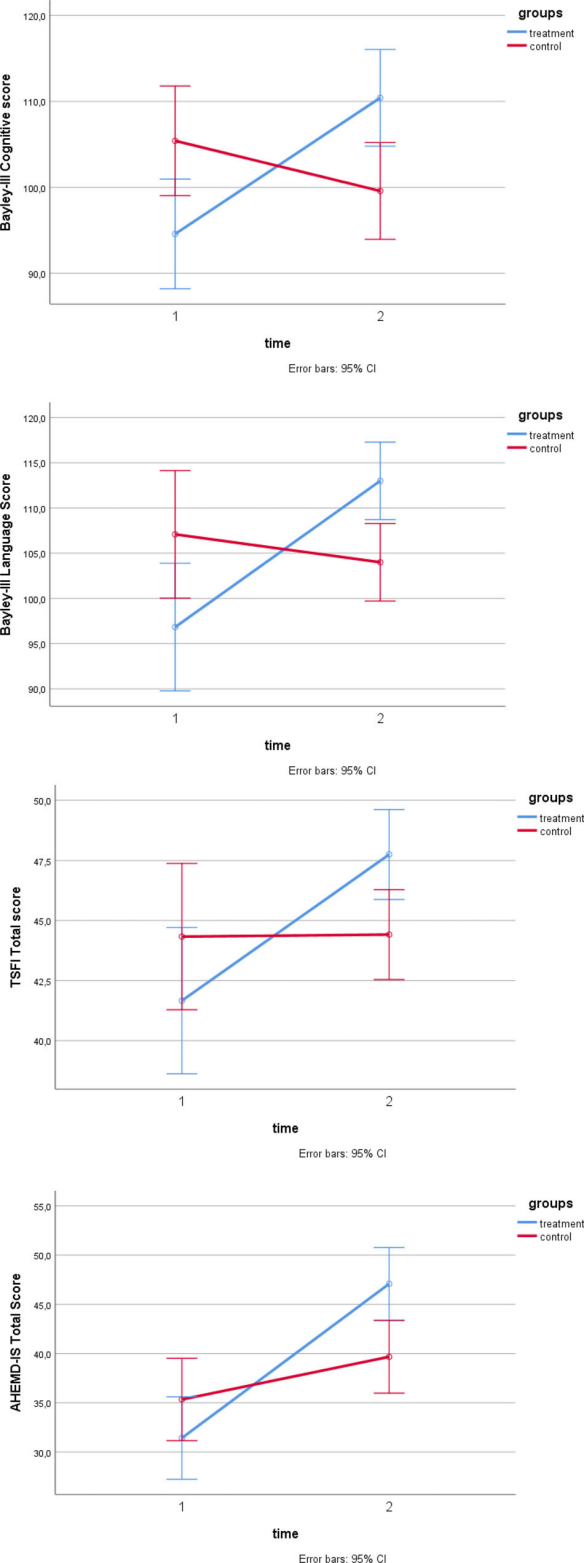
Comparisons of treatment and control groups in terms of Bayley‐III cognitive and language composite score, TSFI total score, and AHEMD‐IS total score.

With the exception of the Bayley‐III cognition and language composite score, there was no difference in baseline values between the groups (*p* > .05, these data are not provided in the tables). At 10 weeks, the interaction effects (time × group) demonstrated significant differences in favor of the SAFE early intervention group for the Bayley‐III cognitive and language composite score, TSFI total score, and AHEMD‐IS total score (*p* < .05) (Table 2). When pairwise comparison was investigated, we listed four comparisons as SAFE group: T2 versus T1, NDT group: T2 versus T1, T1: SAFE versus NDT group, and T2: SAFE versus NDT group. Bayley‐III cognitive score comparisons were, respectively, SAFE group: T2 versus T1 (*p*: .001), NDT group: T2 versus T1 (*p*: .175), T1: (*p*: .021), and T2: (*p*: .010). Bayley‐III language score comparisons were, respectively, SAFE group *p* < .001, NDT group *p*: .391, T1: *p*: .051, and T2: *p*: .005. TSFI total score pairwise comparisons were, respectively, SAFE group *p* < .001, NDT group *p*: .934, T1: *p*: .212, and T2: *p*: .016. AHEMD‐IS total score pairwise comparisons were, respectively, SAFE group *p* < .001, NDT group *p*: .010, T1: *p*: .184, and T2: *p*: .008. Bayley‐III cognitive and language composite score, TSFI total score, and AHEMD‐IS total score were significantly increased only in the SAFE group (*p* < .05) (Figure 2). Nevertheless, there was no difference in the interaction effects (time × group) of the groups in terms of the Bayley‐III motor composite score, COPM Performance score, and COPM Satisfaction score (*p* > .05) (Table 2). There was no difference in baseline and after 10 weeks DASS‐21 subscores and total score in groups (*p* > .05) (Table 3). On the other hand, Bayley‐III cognitive, language, motor composite score and TSFI total score were improved only in the SAFE group (*p* < .05) (Table 2), whereas COPM performance and satisfaction score and AHEMD‐IS score improved in both groups (*p* < .05) (Table 2). There was no difference in DASS‐21 subscores and total scores in both groups in terms of within‐group changes (*p* > .05) (Table 3).

**TABLE 2:** The Bayley‐III, TSFI, COPM, and AHEMD‐IS scores of infants.

Outcome	Group	T1 mean ± SD (95% CI)	T2 mean ± SD (95% CI)	Change mean ± SD	Cohen's *d*	Main effect for group *p* *F* *η* ^2^ *p*	Main effect for time *p* *F* *η* ^2^ *p*	Time × group *p F*		*η* ^2^ *p*
Bayley‐III Cognitive composite score	SAFE	94.58 ± 10.96 (88.20−100.96)	110.41 ± 11.37 (104.78−116.04)	10.83 ± 21.08	0.513	1.0 0 0	.104 2.880 .116	**.001** 13.520	.381	
	NDT	105.41 ± 10.32 (99.04−111.79)	99.58 ± 6.89 (93.95−105.21)	−5.83 ± 10.83	0.538					
Bayley‐III Language composite score	SAFE	96.83 ± 12.41 (89.77−103.89)	113 ± 7.16 (108.71−117.28)	16.16 ± 12.53	1.289	.842 .041 .002	**.015** 6.896 .239	**.001** 14.928		.404
	NDT	107.08 ± 11.12 (100.02−114.14)	104 ± 7.16 (99.71−108.28)	−3.08 ± 11.86	0.259					
Bayley‐III Motor composite score	SAFE	96.16 ± 10.36 (89.14−103.18)	104.66 ± 9.59 (99.06−110.27)	8.50 ±7.70	1.103	.225 1.561 .066	**.007** 8.946 .289	.103 2.899		.116
	NDT	104.16 ± 12.95 (97.14−111.18)	106.50 ± 9.12 (100.89−112.10)	2.33 ± 9.64	0.241					
TSFI total score	SAFE	41.66 ± 5.94 (38.62−44.71)	47.75 ± 1.71 (45.87−49.62)	5.83 ± 5.07	1.149	.834 .045 .002	**<.001** 19.171 .466	**<.001** 18.149		.452
	NDT	44.33 ± 4.05 (41.28−47.37)	44.41 ± 4.07 (42.54−46.28)	1.66 ± 2.77	0.599					
COPM Performance	SAFE	3.90 ± 1.29 (3.18−4.62)	7.48 ± 1.31 (6.35−8.61)	3.57 ± 2.44	1.463	.888 .020 .001	**<.001** 79.150 .783	.541 .386		.017
	NDT	4.21 ± 1.10 (3.49−4.93)	7.32 ± 2.32 (6.19−8.45)	3.10 ± 2.16	1.435					
COPM Satisfaction	SAFE	4.07 ± 1.79 (3.14−5)	7.89 ± 1.19 (6.74−9.04)	3.82 ± 1.96	1.948	.650 .212 .010	**<.001** 62.584 .740	.262 1.326		.057
	NDT	4.29 ± 1.26 (3.36−5.22)	7.14 ± 2.43 (5.99−8.29)	2.85 ± 2.15	1.325					
AHEMD‐IS total score	SAFE	31.41 ± 7.29 (27.23−35.60)	47.08 ± 6.80 (43.38−50.77)	12.33 ± 6.51	1.894	.485 .505 .022	**<.001** 84.979 .794	**<.001** 27.288		.554
	NDT	35.33 ± 6.67 (31.14−39.51)	39.66 ± 5.46 (35.97−43.36)	6.83 ± 6.45	1.058					

*Note*: Data are presented as mean ± standard deviation (SD).

Abbreviations: AHEMD‐IS, Affordances in the Home Environment for Motor Development ‐ Infant Scale; CI, confidence interval; COPM, Canadian Occupational Performance Measure; NDT, neurodevelopmental treatment‐based home program; SAFE, SAFE Early Intervention Approach; TSFI, Test of Sensory Functions in Infants.

*p < .05 for interaction (time × group) by analysis of variance (ANOVA).

**TABLE 3:** Parent mental health baseline and after 10 weeks.

	SAFE (*n*: 12) Median (IQR)			NDT (*n*: 12) Median (IQR)			Mann−Whitney U test
DASS‐21	Pre‐treatment	Post‐treatment	Wilcoxon test *p*	Pre‐treatment	Post‐treatment	Wilcoxon test *p*	*p*
Depression	0.5 (0−2.5)	1 (0.5−3.5)	.07	2 (1−5)	1.5 (1−5)	1.00	.46
Anxiety	1 (1−5)	1.5 (0−3)	.17	1.5 (0−2.5)	1 (0−2)	.06	.51
Stress	2.5 (1−3)	3 (1−4)	.29	4 (3−5)	2.5 (2−3.5)	.07	.95
Total score	5 (2.5−9)	5 (3−9.5)	.38	9 (4−11)	6 (4.5−8)	.06	.54

*Note*: Data are presented as median (IQR).

Abbreviations: DASS‐21, Depression, Anxiety and Stress Scales‐Short Form; NDT, neurodevelopmental treatment‐based home program; SAFE, SAFE Early Intervention Approach.

*p < .05 for within the group by Wilcoxon test and *p < .05 for group differences by Mann−Whitney U test.

### 3.4 Dose of intervention

Twelve SAFE families and 12 NDT families kept complete logbooks, which were gathered throughout the second evaluation period (after 10 weeks). The total dose of therapies was calculated from logbooks. Parents in the SAFE group reported they spent a median of 65 (range: 60–67.5) and a mean of 64.16 min per day, while NDT group parents spent 55 (range: 50–65) and a mean of 64.58 min. There was no difference between groups (*p* = .12).

## 4 DISCUSSION

This study showed significant group‐by‐time interactions in favor of the SAFE group on cognitive, speech and language, and sensory processing in preterm infants with mild to moderate risk factors. The 10‐week SAFE approach produced moderate to significant positive benefits on cognition, speech and language, motor development, sensory processing, goal‐oriented performance, and home enrichment, as seen by the within‐group improvements. On the other hand, goal‐oriented performance and home enrichment increased also in the NDT group after the 10‐week of home program, whereas cognitive, speech and language, motor development, and sensory processing did not change.

### 4.1 Motor, cognitive, and language development

It has been stated that traditional approaches have limited effects on motor and cognitive development, the results of early intervention and rehabilitation approaches, such as COPCA and GAME, have positive results (Blackman, 2002; Dirks et al., 2011; Morgan et al., 2016; Novak et al., 2020; Sgandurra et al., 2014; Spittle et al., 2015). In a randomized controlled study by Blauw‐Hospers et al., the COPCA approach and the traditional program were compared. As a result of the study, no significant difference was observed between motor, cognitive, and language development in the third, sixth, and 18th months. However, the authors noted that functional assessment scores at 18 months were higher in the group treated with the COPCA approach (Blauw‐Hospers et al., 2011; Hielkema et al., 2020). In a randomized controlled single‐blind study published by Morgan et al. in 2016, significant improvement was observed in the areas of motor, cognitive, and functional development in favor of the GAME approach (Morgan et al., 2016). Another early intervention approach using environmental enrichment is SPEEDI. In this approach, the perception‐action model is adopted and it is aimed to explore the movement during the game environment. In the feasibility study conducted by Dusing et al., a significant difference was observed in the areas of motor, language, and cognitive development in favor of the SPEEDI group at the 6th‐month evaluations (Dusing et al., 2015). In a randomized controlled study published by Dusing et al. in 2018, no statistically significant difference was found in the areas of motor and cognitive development, but clinical significance was observed in favor of the SPEEDI group. It was also found that the infants’ problem‐solving skills were significantly higher in the SPEEDI group (Dusing et al., 2018). Our study also showed similar features to recently published studies such as GAME and SPEEDI in that it used enriched environmental principles and collaboration with the family. Sensory integration strategies were used unlike in the studies mentioned. As a result of our study, there was a significant improvement in the SAFE group in Bayley III cognitive, language, and motor development areas. Similarly, there was a significant group time interaction in the cognitive and language development areas in favor of the SAFE group. It was thought that this difference between the groups was due to the creation of a more enriched physical and psychosocial home environment in the SAFE group focusing on communication and interaction with the family members. Similar studies in the literature also show the positive effects of enriched environments on developmental areas (Kavousipor et al., 2021; Miquelote et al., 2012). In a meta‐analysis study published in 2013, it was stated that making home visits during treatment had a positive effect on the cognitive development of children (Filene et al., 2013). Although home visits were made in both treatment groups, environmental enrichment was achieved more in the SAFE group. In addition, the SAFE group created an environment for just‐right challenge for the infants. Self‐initiated movements were encouraged. Also, the variations and diversity of the movement were explained to the families. By using sensory strategies, sensory‐perception‐motor integrity was achieved in the SAFE group. Since the brain is very plastic at this period of life, we tried to use this advantage for a better neurodevelopmental outcome. Also, using the basic principles of interaction and communication in the SAFE approach is a huge advantage for psychomotor development. We believe that all these parameters contributed to significant developmental improvements in the SAFE group. While significant improvement was observed in the SAFE group in motor development areas within‐group measurements, no difference was observed between group time interactions at the end of the treatment. It was thought that this might be because the infants in this group had better motor development scores at the beginning. We could also say that active physiotherapy applied by physiotherapists may have positive outcomes on child motor development. It was observed that families in both groups spent more time on gross motor milestones, such as crawling, cruising, and walking, which contributes to the absence of differences in the field of motor development. At the same time, infants in both groups had a low risk for neurodevelopmental disorders, such as cerebral palsy (CP). It may also have an impact on the outcomes.

### 4.2 Sensory development

In early intervention approaches, such as COPCA, GAME, and SPEEDI, sensory processing problems, which are commonly seen in premature infants, are ignored (Dirks et al., 2011; Dusing et al., 2020; Morgan et al., 2016). With the creation of the SAFE treatment approach, this gap in the literature has been tried to be filled. As part of the SAFE approach, sensory integration principles were used, and sensory strategies and sensory‐motor enriched home environments were created. Studies have shown that sensory problems in infants affect motor and cognitive development (Celik et al., 2018; de Paula Machado et al., 2019). Pekcetin et al. investigated the effectiveness of the sensory strategy and sensory integration interventions in premature infants. According to the study's findings, preterm infants struggle more with sensory processing than term babies do. In addition, it was stated that treatment for 8 weeks of sensory processing increased sensory development in preterm infants (Pekçetin et al., 2016). Dunstan and Griffiths stated in their systematic review study that sensory strategies should be used in home and school environments. It was stated in this review that sensory strategy interventions performed in cooperation with the family in infants or children with sensory processing problems contribute to future developmental outcomes (Dunstan & Griffiths, 2008). In our study, sensory strategies were used as a principle of the SAFE approach. In this framework, sensory and motor‐enriched home environments were created, and strategies for improving sensory processing were used. After 10 weeks of treatment, a significant group time interaction was observed in favor of the SAFE group in the TSFI total score. The results of our study showed that sensory processing problems can be seen in premature infants, and these problems can be overcome or minimalize with sensory strategies and family collaboration. At the same time, we think that positive developments in sensory processing may contribute to the psychomotor and cognitive development of infants. It was concluded that using sensory strategies in natural environments may also affect other development areas, such as motor, social, and speech and language. Therefore, supporting the strong side of the families in this regard in the early stages will positively affect the success of rehabilitation.

### 4.3 Function

Significant improvements were observed in the SAFE and NDT groups in terms of satisfaction and performance in COPM, but no difference was observed between the groups. It was seen that the goals were mostly determined in the areas of fine and gross motor development in this study. The goals mostly consisted of activities, such as “independent walking,” “independent cruising,” and “eating pieces of food on the highchair.” We think that there was no group time interaction between the groups, because the goals were mostly in motor development area. The development in both groups shows that Turkish families spend their time intensively on stages, such as sitting, cruising, and walking, which are important motor development steps for independence in daily life.

### 4.4 Home environment

A sensory and motor‐enriched home environment was created in the SAFE group. The home environment was evaluated with AHEMD‐IS. A significant group time interaction was observed in favor of the SAFE group in the AHEMD‐IS total score. Thanks to the SAFE approach, more focus was placed on play activities in cooperation (interaction and communication) with families. Families developed the activities we suggested and produced different activities. At the same time, we observed that they acquired different types of toys, equipments, and materials during the creation of sensory activities throughout the treatment process. Therefore, we think that the enriched environment (motor, social, cognitive) was better provided in the SAFE group than in the NDT group. Enriched home environment also contributed to the significant improvements in cognitive and language development in the SAFE group. Apart from other studies, SAFE approach created an enriched home environment from the sensory aspects. Supporting sensory processing of preterm infants at 9–10 months of age may also improve the other aspects of development. As a result, a significant difference was also observed in sensory development. We believe that our study will contribute to the literature in this respect.

### 4.5 Parent mental health

In terms of depression, anxiety, and stress, there was no difference between the groups, which was consistent with the GAME approach's findings. Family‐oriented or family‐collaborative approaches in premature infants at risk have important responsibilities to their families. Therefore, it is stated that families may experience intense stress or anxiety (Miles et al., 2007). However, the opposite results have been observed in studies published in recent years (Akhbari Ziegler & Hadders‐Algra, 2020; Balbino et al., 2016). It was observed that the SAFE, which is a family collaborative approach, did not impose extra stress, depression, or anxiety on families. We think that this result of our study is important in that it shows similar features to the studies published in recent years and encourages the use of family collaborative approaches more. It has been demonstrated that it is important for families to be competent in the treatment of their own child, rather than home programs given to families with the logic of exercising in a certain number of time per day, in controlling their stress levels.

### 4.6 Dose of intervention

It is stated that intensively applied motor training programs in premature infants, particularly in newborns at risk of developing CP, may affect the treatment results. In the study of Myrhaug et al., it was shown that intensive treatment programs applied to children with CP improved motor outcomes and functional skills (Jahnsen et al., 2014). In their study, Sakzewski et al. (2014) stated that the duration of treatment is an important parameter in activity‐based and goal‐oriented motor training studies. In our study, the duration of treatment was noted daily by the families. At the end of the treatment, it was observed that there was no difference between the groups in terms of treatment duration. Based on the studies in the literature, we see that intensive treatments affect the developmental stages. However, in our study, intervention time for each group was similar. We think that this is important in that it does not affect neurodevelopmental outcomes.

### 4.7 Limitations

The limitation of our study was that the premature infants included in the study had a mild to moderate risk of developing neurodevelopmental problems, such as CP. The inclusion of infants at a better level may have enabled developmentally positive results. We think that our results should be supported by further studies that include infants with high risk for CP. Conducting research with more premature infants in future studies is important in terms of increasing the applicability of the SAFE early intervention approach. In this study, the effectiveness of the SAFE early intervention approach in mild to moderate preterm infants at risk with adjusted ages between 9 and 10 months was examined. There is also ongoing research about the implementation of the SAFE approach in different ages for infants. We think that investigating the effectiveness of the SAFE early intervention approach in future studies, especially in premature and term‐born infants without fidgety movements, is important in terms of its contribution to the literature.

## 5 CONCLUSIONS

Our study suggests that for preterm infants at 9–12 months of age, SAFE is a clinically feasible intervention and more effective than NDT to improve cognitive, speech and language, and sensory functions of preterm infants in an enriched home environment. It was observed that SAFE early intervention approach did not cause a state of stress, depression, or anxiety that would adversely affect the mental health of the families and/or caregivers. SAFE is a promising new early intervention approach for preterm infants.

## AUTHOR CONTRIBUTIONS

Umut Apaydın: Conceptualization, analysis, and writing of the first draft of the manuscript. Ramazan Yıldız: Data collection and tracking of the treatment programs. Ayşe Yıldız: Data collection and tracking of the treatment programs. Şebnem Soysal Acar: Conceptualization, performing the Bayley‐III assessment, and critical editing of the manuscript. Kıvılcım Gücüyener: Conceptualization and critical editing of the manuscript. Bülent Elbasan: Conceptualization and critical editing of the manuscript. All the authors approved the final version of the manuscript.

## FUNDING INFORMATION

This research did not receive any specific grant from funding agencies in the public, commercial, or not‐for‐profit sectors.

### PEER REVIEW

The peer review history for this article is available at https://publons.com/publon/10.1002/brb3.3199.

## CLINICAL TRIAL NUMBER

NCT04889846.

## Supporting information

Supporting information

## Data Availability

The data supporting this study's findings are available from the corresponding author upon reasonable request.
