# Supplementary material for: Short‐term effects of SAFE early intervention approach in infants born preterm: A randomized controlled single‐blinded study
Source: Brain Behav. 2023 Aug 3;13(10):e3199. doi: 10.1002/brb3.3199 (PMC10570479; doi:10.1002/brb3.3199)
Supplement: Supplementary file 1 — Supporting information [file BRB3-13-e3199-s001.docx]

1. **Neurodevelopmental Therapy (NDT)-based home program (control group)**

Within the scope of this study, an NDT-based home program was given to the infants in the control group according to their corrected ages and current functional levels. In this context, appropriate activities were recomended to families. Families were asked to do these activities and variations every day for 10 weeks. It was ensured that families' compliance with the program was monitored every week by phone call or via the WhatsApp program. In addition, families were asked to keep a diary and note the duration of their activity. Two visits were made to the homes of the families in the control group. The family's questions about the program were answered. The home environment was evaluated. The applications and objectives of the NGT-based physiotherapy programs, which are determined and used in the light of the motor movements that the baby can perform, are listed below:

- Providing regulation of muscle tone with massage and intramuscular stretching in infants with increased muscle tone,
- Hands on facilitation while sitting independently and developing voluntary hand-eye coordination, holding, grasping, and trunk control in this position,
- Developing midline orientation by reaching and touching objects in the midline in the side-sitting position,
- Optimising trunk control by trunk elongation, lateral flexion, weight-bearing, and reaching in the side-sitting position,
- Facilitation of rolling, sitting, standing, and walking in accordance with the level of motor development,
- The use of the hands for both support and activity in supported and unsupported sitting positions, and facilitation of the sitting position with trunk extension,
- Ensuring proper posture in the supported standing position, facilitating weight-bearing, and trunk extension,
- Cruising
- Making transitions between different heights while cruising,
- Making squat at different knee angles in supported standing
- Walking activities
- Fine dexterity activities (for example, trying to put 2 cubes on top of each other and placing small balls in the bottle),

During these activities, families were explained in detail how facilitation should be done. In addition, families were taught how to make hand contacts.


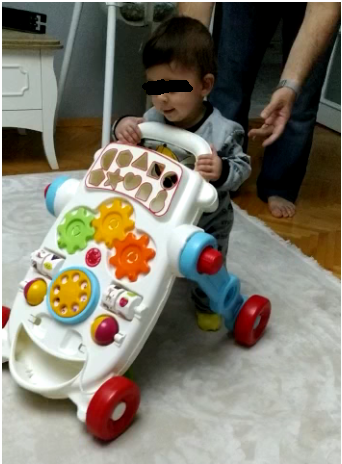

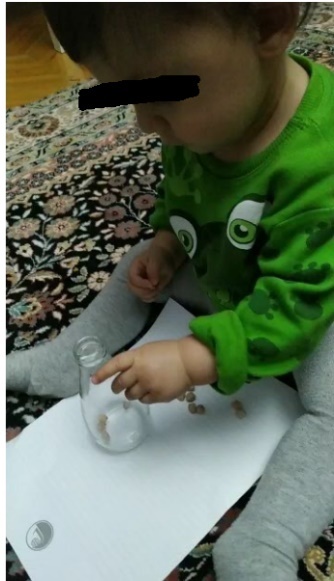

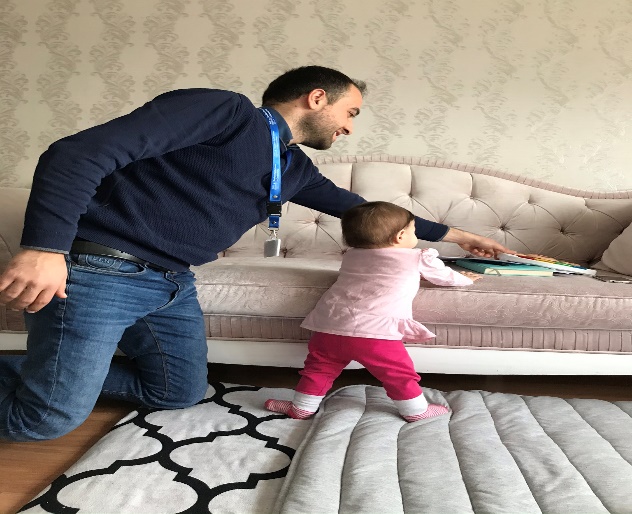


Picture 1. NGT-based physiotherapy and rehabilitation program

1. **SAFE Early Intervention Approach**
   1. **Theoretical framework**

The SAFE early intervention approach is family-centered and focuses on activity-based motor training and sensory strategies in an enriched environment. The theoretical and clinical application framework of the intervention was developed in the Developmental Physiotherapy and Pediatric Rehabilitation Unit of XXX University Faculty of Health Sciences, Department of Physiotherapy and Rehabilitation. The early intervention approach is carried out in the child's home environment, and a collaboration between the physiotherapist and family is established to implement the program. The SAFE approach is based on neuronal group selection theory (NGST) and motor learning principles. At the same time, this approach includes ecological model principles in its practical applications in the natural context. The NGST argues that normal motor development is characterized by adaptive variability and variation. There are strategy repertoires for each motor function, and the brain selects and processes the most appropriate one from these repertoires. Initially, the infant cannot choose from the repertoire of strategies best suited to the situation, but gradually, over time, the ability to choose the most appropriate strategy for each situation develops. The selection process relies on active trial-and-error experiences and associated sensory information in their natural context. This means that the baby's self-generated sensorimotor experience plays a very important role in psychomotor development. When there is an early lesion in the brain, the ability to change motor behavior variability is restricted. In addition, the impairment in the processing of senso-motor information and the decrease in the repertoire prevent the child from finding the best motor behavior. Therefore, infants at risk may choose a different motor pathway than their typically developing peers. Because of the impairment in the processing of senso-motor information, the infant must experience more active senso-motor experiences than a typically developing peer. For these reasons, within the scope of SAFE, plenty of active trial and error are included in various conditions and in accordance with the NGST principles. The motor learning process is supported with “hand-off” methods, and the child is given the opportunity to explore the activities of daily life by making just right challenge in different environments. NGST recognizes that the brain is shaped by signals from the body and interaction with the environment during nataland postnatal development. In this context, it is aimed to increase neuroplasticity and support motor function with social, motor, and sensory stimulations in an enriched environment with the SAFE approach.

- 1. **Practical framework**

Appropriate activities within the scope of the SAFE approach were explained to the families. Families were asked to do these activities every day for 10 weeks. It was ensured that families' compliance with the program was monitored every week by phone call or via the WhatsApp program. In addition, families were asked to keep a diary and note the duration of their activity. At least 1 visit was made to the homes of the families in the treatment group. The home environment was evaluated during this visit. In order to create an enriched home environment, families were informed about the toys and materials that could be obtained. The family's questions about the program were answered. Within the scope of the SAFE approach, examples of activities suitable for the family's goals and the performance of infants are as follows:

- For the tactile system, the selection of textured toys and the creation of the playground from different materials (creating the texture of the surfaces on which the child crawls, cruises, or walks from different floors such as jagged, rough, and hairy),
-
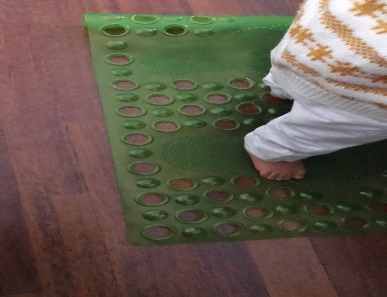

- Crawling, cruising, and walking activities on soft ground,
- Swinging the baby in a blanket at different speeds and directions for the vestibular system, performing targeted activities for the vestibular system on the ball and roller


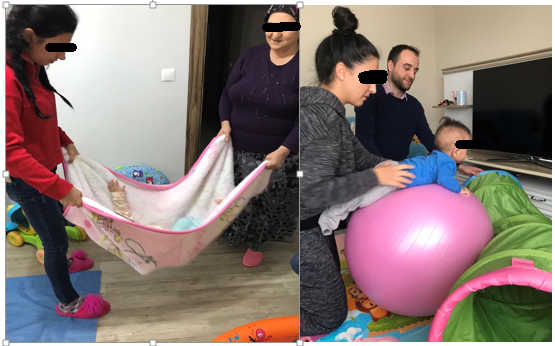


- Linear rhythmic oscillations for the vestibular system
- Playing with heavier toys in the prone an/or crawling position for vestibular and proprioceptive sensory development,
- Playing hiding games (for example, throwing a blanket on the child and getting rid of it),
- Supporting functional skills with substances such as whipped cream, yogurt, and shaving foam for the sense of tactile on the highchair,
- Spending time in the sensory tunnels (in cases where tunnels cannot be purchased, creating a tunnel by the sofa with pillows, and setting a target at one end of the tunnel),


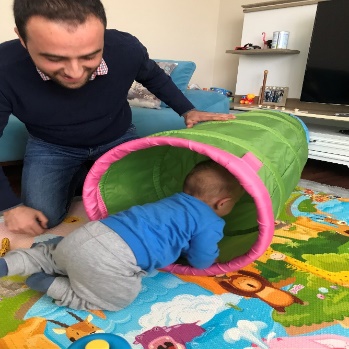


- Spending time in the ball pool and giving tactile stimulus, teaching prone and side sitting positions, position transitions while sitting in the ball pool,
- Spending time with brighter and illuminated toys for the sense of vision, reaching for toys and weight-bearing,
- Spending time with toys with different frequencies and intensities for the development of the sense of hearing,
- Creating an environment of just right challenge in children who have succeeded in motor activity; for example, creating uneven surfaces and performing climbing and descending activities on this ground,


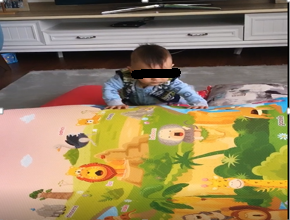


- Creating a ramp from pillows to the edges of the sofa and climbing and descending activities e,
- Reading books suitable for supporting cognitive development,
- Limitation of passive television viewing (A maximum of 30 minutes of television viewing per day is allowed up to the age of 18 months),
- Activities that provide fine grasping (trying to stack 2 cubes on top of each other or throwing objects such as rice and chickpeas into the bottle),
- Adding sound stimuli such as music to the environment when performing goal-oriented motor activities in infants who are extremely disturbed by noisy environments.

Within the scope of the SAFE early intervention approach, detailed information was given to the families about how to create a motor and sensory enriched home environment. In addition, detailed explanations were made about the motor development levels of babies. The families were told how a motor movement was performed by the child. During motor movements, families were asked to give their children the opportunity to do trial and error. They were asked to avoid hand contact as much as possible. The importance of the baby's self-initiated movement was explained to the families.
